# Tai Chi Chuan vs General Aerobic Exercise in Brain Plasticity: A Multimodal MRI Study

**DOI:** 10.1038/s41598-019-53731-z

**Published:** 2019-11-21

**Authors:** Lei Cui, HengChan Yin, ShaoJun Lyu, QiQi Shen, Yuan Wang, XiuJuan Li, Jing Li, YunFei Li, LiNa Zhu

**Affiliations:** College of P. E. and Sports, Beijing Normal University, Beijing, 100875 China

**Keywords:** Neuroscience, Psychology, Health care

## Abstract

This study contrasted the impact of Tai Chi Chuan and general aerobic exercise on brain plasticity in terms of an increased grey matter volume and functional connectivity during structural magnetic resonance imaging (sMRI) and resting-state functional magnetic resonance imaging (rs-fMRI), explored the advantages of Tai Chi Chuan in improving brain structure and function. Thirty-six college students were grouped into Tai Chi Chuan (Bafa Wubu of Tai Chi), general aerobic exercise (brisk walking) and control groups. Individuals were assessed with a sMRI and rs-fMRI scan before and after an 8-week training period. The VBM toolbox was used to conduct grey matter volume analyses. The CONN toolbox was used to conduct several seed-to-voxel functional connectivity analyses. We can conclude that compared with general aerobic exercise, eight weeks of Tai Chi Chuan exercise has a stronger effect on brain plasticity, which is embodied in the increase of grey matter volume in left middle occipital gyrus, left superior temporal gyrus and right middle temporal gyrus and the enhancement of functional connectivity between the left middle frontal gyrus and left superior parietal lobule. These findings demonstrate the potential and advantages of Tai Chi Chuan exercises in eliciting brain plasticity.

## Introduction

Brain plasticity refers to the ability of human brain structure and function to be continuously modified and reconstructed with changes in the internal and external environment. It is an important physiological characteristic of the nervous system, as well as the physiological basis for the adaptive changes in human psychology and behaviour and is consistent throughout the life of an individual. Today, there is accumulating evidence that the human brain also continues to be shaped by experience throughout adulthood^1–4^. These adaptive changes have been shown to take place on structural and functional level^5–7^. Although early experimental studies were mainly performed in animals^8–10^, technical developments, especially in the field of MRI, have enabled the non-invasive observation of structural and functional reorganization in the human brain.

Grey matter volume (GMV) refers to the volume of grey matter between the white matter surface and the soft membrane surface of the human brain, which is roughly positively correlated with the number of neurons in the brain and has become a major indicator for studying the plasticity of the brain structure. Resting-state functional connectivity (FC), which is defined as the synchronization of brain regions with each other^11,12^, has attracted particular attention for its ability to measure correlations in neural activity between distant brain regions. These correlations are of great interest to the medical community because an increasing number of pathologic conditions appear to be reflected in functional connectivity between particular brain regions^13–15^. Resting-state functional connectivity closely resembles patterns of anatomical connectivity through white matter (WM) fibre pathways and covaries with cortical GMV^16^.

Because brain plasticity allows people to adapt to changing environments and needs, how to strengthen the mechanisms of brain plasticity to prevent cognitive decline in human lifespan, and promote memory, learning and recovery after brain injury has become a focus of research. In recent years, evidence from both human and animal studies has suggested that physical activity and exercise promote neuroplasticity, often accompanied by improvements in cognitive function^17^. Regular exercise is a practical way to enhancing brain plasticity^18–25^.

Tai Chi Chuan (TCC), a multimodal mind-body exercise integrating gracefulness, mindfulness, and gentleness, is a form of traditional Chinese exercise that involves physical activity, cognitive control, and social interaction when practised in a group. It combines the coordination of slow movements with mental focus, deep breathing, and relaxation. It can be practised without special facilities or expensive equipment and can be performed either individually or in groups. The benefits of practising Tai Chi have been reported in cognitive performance^26,27^ and motor functions, such as postural control^28^, fall prevention^29^, muscle strength, and agility^30^. Previous studies have shown that long-term TCC practice can improve the fractional amplitude of low-frequency fluctuations^31–33^, modulate functional connectivity of the cognitive control network^34^, optimise locally functional organization^35^ and changes in brain structures^36^ of older adults. However, above studies have explored the neural mechanism with a single imaging technique, which cannot provide complete information about the plasticity of the brain, and the anatomical structure of the brain or functional information reflected by each imaging technique is limited. And whether TCC’s effect on brain plasticity is superior to general aerobic exercise (AE) is remain unclear and worth further discussion. Bafa Wubu of Tai Chi is a new set of TCC^37^, including simple structure, reasonable quantity, and rich connotation, which is easy to learn and practice and now is being popularized in China. It is based on the existing 24-style TCC, refining and organizing systematically from the Bafa Wubu techniques which is the essence of various types of TCC.

In summary, we used a multimodal MRI approach to investigate whether long-term TCC practice, represented by Bafa Wubu of Tai Chi, could induce regional structural GMV and functional connectivity (FC) changes and whether the effect is superior to aerobic exercise.

## Results

### Sample characteristics

There were no significant differences between the three groups in gender, age, handedness, average years of education, and body mass index (BMI) (Table 1).

**Table 1:** Sample characteristics.

Items	TCC	AE	Control	F	p
	M(SD)	M(SD)	M(SD)		
Gender (Male/Female)	2/10	2/10	2/10	—	—
Age (years)	21.83 (2.48)	21.92 (2.28)	21.75(2.45)	**0.014**	**0.986**
Handedness (Left/Right)	12/0	12/0	12/0	—	—
Education (years)	16.33 (2.23)	16.41 (2.27)	16.33 (1.50)	**0.007**	**0.993**
BMI (kg/m^2^)	20.21 (2.54)	19.15 (2.06)	21.71 (2.96)	**3.07**	**0.06**

### GMV results

**Figure 1:**
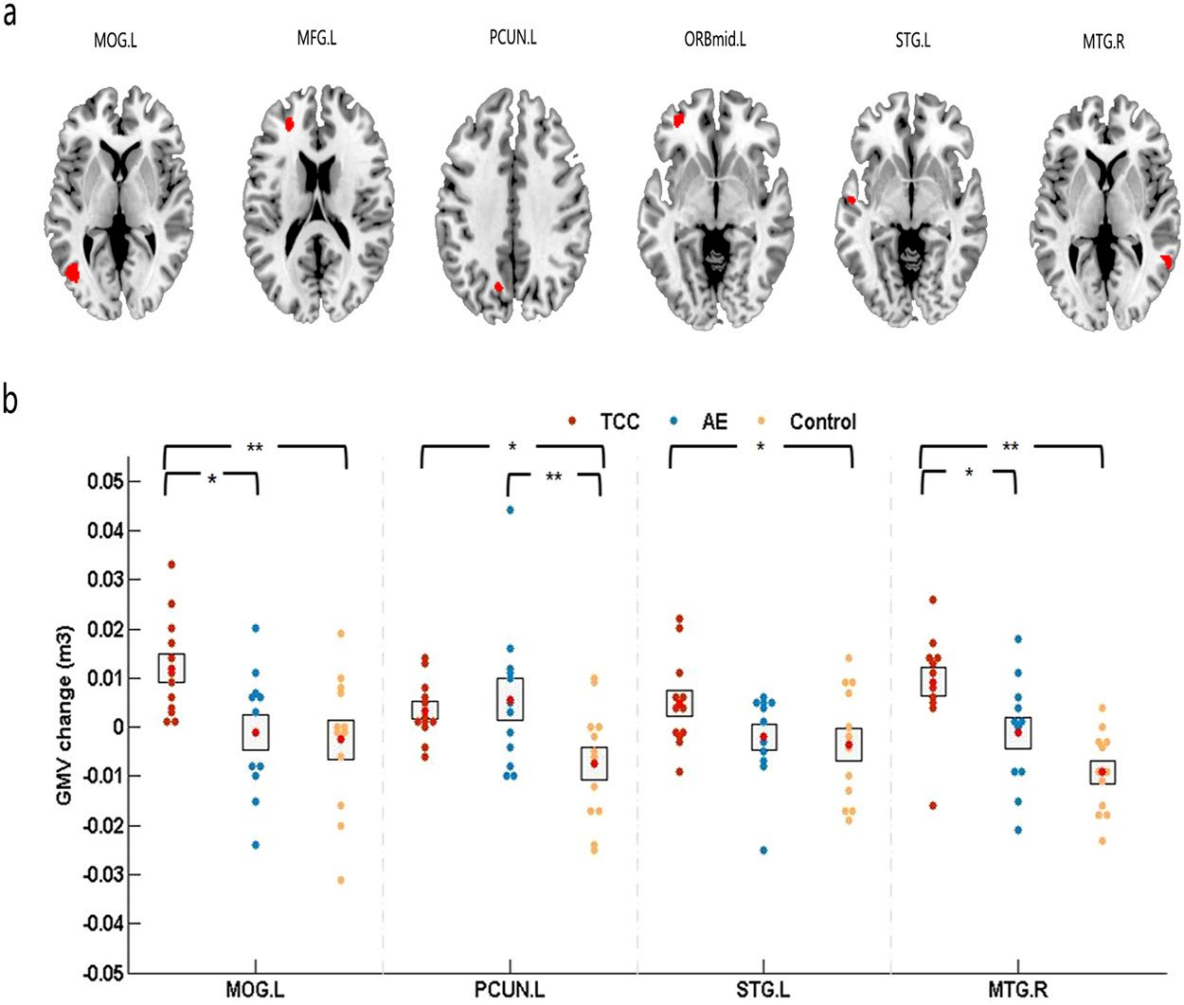
GMV results. Changes in grey matter (GM) during 8 weeks of TCC and AE exercise. (**a**) The results of repeated-measures ANOVA are presented on the axial slices of the grey matter template (MNI); FWE corrected at p < 0.05. Abbreviations: MOG.L-left middle occipital gyrus, MFG.L-left middle frontal gyrus, PCUN.L-left precuneus, ORBmid.L-left middle frontal gyrus, orbital part, STG.L-left superior temporal gyrus, MTG.R-right middle temporal gyrus. (**b**) The results of post hoc statistical analysis of GMV changes (post minus pre). *means p < 0.05, **means p < 0.01.

A 3 Group (TCC, AE, control) × 2 Time (pre, post) repeated-measures ANOVA on the GMV yielded six significant interactions [the threshold for significant changes was set to *p* = 0.05 whole brain cluster-level FWE corrected with a cluster size set to 50, with a building threshold of *p* = 0.001 uncorrected on voxel level], including (Fig. 1a): left middle occipital gyrus (MOG.L; cluster size: 307; peak MNI coordinate: −37.5, −73.5, 7.5; F = 53.2243); left middle frontal gyrus (MFG.L; cluster size: 161; peak MNI coordinate −25.5, 42, 18; F = 53.1549); left precuneus (PCUN.L; cluster size: 51; peak MNI coordinate −12, −67.5, 39; F = 41.9011); left middle frontal gyrus, orbital part (ORBmid.L; cluster size: 42; peak MNI coordinate: −34.5, 45, −6; F = 39.6032); left superior temporal gyrus (STG.L; cluster size: 76; peak MNI coordinate: −55.5, −9, −4.5; F = 38.5559); right middle temporal gyrus(MTG.R; cluster size: 110; peak MNI coordinate: 58.5, −51, 4.5; F = 37.4611).

Pre- and post-exercise comparisons of GMV among the three groups showed that after 8 weeks, GMV was significantly increased in the MOG.L (p < 0.01), PCUN.L (p < 0.05), STG.L (p < 0.01) and MTG.R (p < 0.01) in the TCC group compared to the control group. In the AE group, there was a significant increase in GMV in the PCUN.L (p < 0.01) compared to the control group. Compared with the AE group, the TCC group was associated with a significant GMV increase in the MOG.L (p < 0.05) and MTG.R (p < 0.05). (Fig. 1b).

### Resting-state functional connectivity results

**Figure 2:**
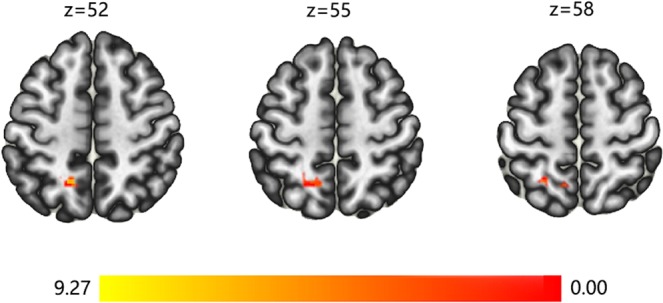
FC results. The results of resting-state functional connectivity analysis are presented on axial slices of the grey matter template (MNI). Yellow and red indicate brain regions that showed significant FC increase (post > pre) in the Tai Chi Chuan group. The threshold for significant changes was set to p < 0.05 cluster mass-level FWE corrected with a cluster building threshold of *p* = 0.001 uncorrected on voxel level.

Seed-based (MOG.L, MFG.L, PCUN.L, ORBmid.L, STG.L, MTG.R) FC analysis was performed. When MFG.L was set as the seed, we found significant FC increases in the left superior parietal lobule (SPL.L; cluster size: 52; peak MNI coordinate −12, 54, 54; mass = 279.47; mass p-FWE = 0.027) in the Tai Chi Chuan group after the 8-week practice (Fig. 2). No significant functional connectivity differences were observed in the AE and control groups.

## Discussion

To the best of our knowledge, this study is the first to systematically contrast the impact of TCC and AE on brain structure and functional connectivity. We found that the GMV of the left MOG, left PCUN, left STG and right MTG significantly increased in the TCC groups compared with the control group after 8 weeks of practice. The GMV of the left PCUN also significantly increased in the AE group compared with the control group after 8 weeks of practice. Interestingly, we also found that 8 weeks of TCC practice significantly increased the GMV of the left MOG, left STG and right MTG compared with AE practice. And TCC practice increases the FC between MFG.L and SPL.L.

Both TCC and AE changed the GMV to some extent. This converges with prior studies showing that exercise intervention can indeed change brain plasticity, including GMV and functional activity^38,39^. Longitudinal brain imaging studies revealed a pronounced degree of training-induced structural plasticity in the adult human brain. Fast-evolving structural brain alterations^40–42^ are more likely related to the formation and proliferation of dendritic spines and axonal varicosities. Several studies have also found that Tai chi can change brain function and grey matter volume in older adults^43,44^ and TCC practitioners are significant differences with subjects who have no physical exercise in cortical thickness and regional homogeneity. The PCUN belongs to the default model network (DMN), which includes functions such as autobiographical memory retrieval, considering the perspective of others, and envisioning the future^45^ and is associated with meditation, higher levels of body representation and self-related processing, and attention transfer^46–48^. A recent study found that stress-linked PCUN cortical thickness represents a candidate prospective biomarker of adolescent depression^49^. Structural changes in the PCUN are associated with increased integration of internal and external feelings, which facilitates the individual’s awareness of the present moment and forms a new perspective of the self. These results suggest that TCC and general AE may have benefits in memory retrieval, meditation, attention to depression and other aspects.

Compared with the AE, TCC significantly increased the GMV of the left MOG, left STG and right MTG. The occipital lobe is highly correlated with basic cognitive processes such as visual search and visual attention^50^. Previous imaging studies have shown the importance of the MOG in memory retrieval^51^, especially associative memory^52^. The STG has been found to be sensitive to emotional information and is involved in mentalizing, especially during awareness or speculation of intention or goal-directed behaviour of others or themselves no matter the biological motions or head and body motion^53–55^. The MTG has been supposed to play an important role in the cortical networks sub-serving memory processing^56–58^. Previous studies found the reduced volume and activation in the medial temporal lobe have been linked to cognitive decline in older adults^59^. At the same time, we found that TCC practice increases the FC between MFG.L and SPL.L. The MFG plays an important role in episodic retrieval^60,61^. MFG and SPL belong to the cognitive control network (CCN). Increased FC between MFG and SPL might reflect changes in neural activity associated with more elaborate memory retrieval and more effective cognitive control in the TCC group. Specifically, evidence suggests that TCC exercise alters resting-state synchrony and causes more mature, efficient patterns of brain activation^62,63^. This result also prompts us that the individual white matter (WM) fibre pathway may also be changed in TCC practice. A previous study showed that FC covaries with GMV, and it is highly correlated with the anatomical connectivity through WM fibre pathways. WM are mainly made up of axonal fibre connections, which can transmit nerve information between different GM regions. Motor learning can induce WM changes in the frontal and parietal lobes of adults, and the changes in WM are spatially similar to GM changes. These suggests that the FC increase between MFG.L and SPL.L may be associated WM alterations.

The special changes of GMV and FC in TCC group may be related to the principles of TCC practice. Compared with general AE, TCC is a multimodal mind-body exercise, which emphasizes the importance of experiencing a wide range of one’s own internal sensations as well as external phenomena outside the body and has be has benefits of general AE and cognitive training. The exercisers are required to complete cognitive activities such as visual space processing, motion recalling and task shifting while doing the movements. TCC movements are continuous from the beginning to the end, and from one posture to the next, in a complete integrated circle. Within this circle movement are many concealed changes: emptiness and fullness, strength and softness, movement forward and backward, action and serenity^64^. The movements emphasis the exercise of mind and consciousness. The harmonious and unified category of Yin and Yang of TCC is build based on the following unification of the contradiction between the opposite movements, like the forward and backward of human body, the rise and fall of one’s centre of gravity, the static and dynamic of one’s whole body and part, the opening and closing of chest, shoulder, elbow, hands, hip, knee and feet, the lengthen and bending of the spine, the shaking of one’s body, the virtual and actual of TCC styles, the rigidity and softness of the movement, etc. Previous studies, which provide support for this study, found that complex exercise can promote cognitive function more than simple repetitive exercise. Moreover, TCC exercise required smooth movement, easy breathing, even speed, integration of hardness and softness, unity, agility and calmness. Overall coordination of “Yi, Qi and Xing” should be emphasized in the practice process, making an organic combination of consciousness, breath and movement through the “calm mind and relaxed body”. Each action can be guided by Yi (mind) with rapt attention to complete the movement in a correct and full manner. Therefore, regular TCC exercise improves their perception of movement information, memory retrieval, cognitive control and other aspects. In the future, relevant cognitive tasks can be combined to verify these aspects. Exploring the effects of different sets of TCC and different moves of TCC on brain plasticity any further, to determine the optimal program of TCC exercise.

In summary, we found that compared with aerobic exercise, 8 weeks of TCC exercise has a stronger effect on brain plasticity, which is embodied in the increase of grey matter volume in MOG.L, STG.L and MTG.R and the enhancement of functional connectivity between MFG.L and SPL.L. These findings demonstrate the potential and advantages of TCC exercises in eliciting neuroplasticity.

There are several limitations in the present study. In neuroimaging, the reliability of the measures which are employed in experiments can vary substantially. Morphological measures are known by the highest reliability in MRI^65^. We not only focus on grey matter volume to determine changes in brain structural morphology, but also on FC to monitor fluctuations in the spontaneous brain activities. However, the reliability of FC is still in dispute^66^. And these results suggest a possible more positive effect of TCC on brain plasticity which compare with the effect of general AE. But the interpretation should be cautious because of the sample size and intervention duration. There may be more subtle changes in neuronal plasticity. Consequently, larger sample size, and deeper exercise duration studies are warranted to confirm the results. There is no behavior measurement to determine relationship between the imaging result and behaviour. Thus, studies can determine the advantages of TCC exercises in behaviour and find the relationship between behaviour and brain plasticity in the future.

## Methods

### Participants and study design

Participants were grouped into three groups matched on age, gender, and years of education. Three groups were randomly assigned to the TCC, AE or control group. Individuals were assessed with a structural MRI and rs-fMRI scan before and after the training period. We tested a total of 36 college students. All subjects were right-handed, with no history of psychiatric or neurological disease. All participants provided written informed consent and were paid for their participation. This study was approved by the Institutional Review Board of the National Key Laboratory of Cognitive Neuroscience and Learning. The research was conducted in compliance with Declaration of Helsinki.

### Exercise intervention and control procedures

The Tai Chi Chuan intervention group (TCC) received three weekly sessions of Bafa Wubu of Tai Chi group training for 8 weeks. Bafa Wubu of Tai Chi is systematically refined and sorted out by the General Administration of Sports of China on the basis of the existing 24 Form Tai Chi, revolving around the most common and core techniques of “Bafa Wubu of Tai Chi”, namely the eight hand techniques of “Peng (warding off), Lu (rolling back), Ji (pressing), An (pushing), Cai (pulling down), Lie (splitting), Zhou (elbowing) and Kao (shouldering)”, and the five footwork of “Jin (advancing), Tui(retreating), Gu (shifting left), Pan (shifting right) and Ding (central equilibrium)”, thus forming a set of Tai Chi routines for popularization characterized by culture, fitness and simplicity. Each training session consisted of 5 min of warmup, 50 min of continuous sequential practice of learned forms, and 5 min of cool-down. Using the Polar Watch (Polar Electro Oy, Kempele, Finland) to monitor participants’ heart rates during the exercise sessions, we found that the intensity of the 50-min continuous TCC practice reached approximately 67.995 ± 1.385% (range = 66.61% to 69.38%) of the individual participants’ age-predicted maximal heart rates (HRmax) on average and thus could be considered moderate intensity (60% to 69% HRmax) endurance exercise according to the classification of American College of Sports Medicine^43^.

The general aerobic exercise group (AE) received three weekly sessions of brisk walking group training for 8 weeks. Each training session consisted of 5 min of warmup, 50 min of continuous sequential practice, and 5 min of cool-down. As in the TCC group, using the Polar Watch to monitor participants’ heart rate during the exercise sessions, we found that the intensity of the 50-min continuous AE practice reached approximately 68.28 ± 1.64% (range = 66.64% to 69.92%)of the individual participants’ age-predicted maximal heart rates (HRmax) on average and thus could be considered moderate intensity (60% to 69% HRmax) endurance exercise.

The control group was instructed to maintain their original daily routines and physical activity habits and to not receive any new or additional exercise interventions.

### Mri data acquisition

For each participant, we applied sMRI (5 min) and fMRI scan (8 min) each in the before and after intervention periods. Images were acquired on a 3.0T MRI system (Siemens Magnetom Prisma; Erlangen, Germany) with a 64 channel head coil in Beijing Normal University Imaging Center for Brain Research.

High resolution three-dimensional T1-weighted magnetization-prepared rapid gradient-echo images were acquired^67^ [repetition time (TR) = 2530 ms, echo time (TE) = 2.98 ms, inversion time = 1100 ms, flip angle (FA) = 7°, slice thickness = 1 mm, 192 sagittal slices, voxel size = 0.5 × 0.5 × 1 mm, field of view (FOV) = 192 × 256 × 256 mm].

Functional images were obtained using an echo-planar sequence sensitive to blood oxygenation level-dependent contrast^67^ (TR = 2000 ms, TE = 30 ms; FA = 90°, slice thickness = 3.5 mm, 33 axial slices, voxel size = 3.5 × 3.5 × 3.5 mm, FOV = 224 × 224 × 138 mm, 240 volumes). The participants were instructed to keep their eyes opened without falling asleep and to move as little as possible. As assessed by a questionnaire, no subjects reported falling asleep during the scanning or being uncomfortable during or after the procedure.

### Data analysis

#### T1 image preprocessing

Imaging data preprocessing was implemented using VBM8 (http://dbm.nero.uni-jena.de), which is based on Statistical Parametric Mapping 8 (SPM 8: http://www.fil.ion.ucl.ac.uk/spm), including the following conventional steps: (1) extraction of brain tissue information and removal of skull, scalp and other non-imaging data, (2) affine transformation of all grey matter images into the ICBM152 grey matter template, resulting in the generation of a custom grey matter template with a resolution of 2 × 2 × 2 mm, (3) registration of the subjects’ grey matter images into a custom grey matter template and smoothing with a Gaussian function with a half-width and full height of 3 mm.

#### Resting-state fMRI image preprocessing

Imaging data preprocessing was implemented using GRETNA (https://www.nitrc.org/projects/gretna), which is based on Statistical Parametric Mapping 12 (SPM 12: http://www.fil.ion.ucl.ac.uk/spm), including the following conventional steps^67^: (1) discarding the first ten time points, allowing for signal equilibrium and adaptation of the participants to the scanning noise, (2) compensation of systematic slice-dependent time shifts, (3) correction for head movement with rigid body translation and rotation parameters, (4) normalization into Montreal Neurological Institute (MNI) space using unified segmentation on T1-weighted images and reslicing into 3-mm cubic voxels, (5) spatial smoothing with a 4-mm full-width at half-maximum Gaussian kernel, (6) removing the signal trend with time linearly, (7) bandpass (0.01–0.08 Hz) filtering to decrease physiological noise, and (8) regression of nuisance variables including head motion parameters(Friston-24), the white matter signal averaged from the deep cerebral white matter(WM) and the cerebrospinal fluid(CSF) signal averaged from the ventricles to further reduce non-neuronal contributions. All data used in this study satisfied the criteria of spatial movement in any direction <2 mm translation or 2° rotation.

#### GMV analysis

GMV analysis was performed with the SPM8 toolkit (SPM 8: http://www.fil.ion.ucl.ac.uk/spm) test AAL116, which determined the brain areas where significant grey matter volume differences were found between each group. A 3 Group (TCC, AE, control) × 2 Time (pre, post) repeated-measures ANOVA on the GMV yielded six significant interactions (the threshold for significant changes was set to *p* = 0.05 whole brain cluster-level FWE corrected with a cluster size set to 50, with a building threshold of *p* = 0.001 uncorrected on voxel level). The REST toolkit (http://restfmri.net/forum/index.php? Q = rest) was used to extract the grey matter volume values in the significantly different brain regions of each subject, followed by post hoc statistical analysis in SPSS25.0.

#### FC analysis

FC analyses were carried out using the CONN functional connectivity toolbox (http://www.nitrc.org/projects/conn). The seeds for the voxel-based functional connectivity analysis were the brain areas with significant changes in grey matter volume. The correlation coefficient between the ROI time series and the AAL116 brain region time series was calculated, and this correlation was called the functional connectivity. The threshold for significant changes was set to p < 0.05 cluster mass-level FWE corrected with a cluster building threshold of *p* = 0.001 uncorrected on voxel level.
